# Prospective Study on Individualized Dose Adjustment of Tolvaptan Based on Urinary Osmolality in Patients With ADPKD

**DOI:** 10.1016/j.ekir.2024.01.020

**Published:** 2024-01-12

**Authors:** F.J. Roca Oporto, C. Andrades Gómez, G. Montilla Cosano, A. Luna Aguilera, José L. Rocha

**Affiliations:** 1 Unidad de Gestión Clínica Nefrología, Hospital Universitario Virgen del Rocío, Sevilla, Spain; 2 Nefrología, Departamento Medicina, Universidad de Sevilla, Sevilla, Spain

**Keywords:** autosomal-dominant polycystic kidney disease, estimated glomerular filtration rate, tolvaptan, urinary osmolality

## Abstract

**Introduction:**

Tolvaptan has been shown to reduce renal volume and delay disease progression in autosomal-dominant polycystic kidney disease (ADPKD). However, no biomarkers are currently available to guide dose adjustment. We aimed to explore the possibility of individualized tolvaptan dose adjustments based on cut-off values for urinary osmolality (OsmU).

**Methods:**

This prospective cohort study included patients with ADPKD, with rapid disease progression. Tolvaptan treatment was initiated at a dose of 45/15 mg and increased based on OsmU, with a limit set at 200 mOsm/kg. Primary renal events (25% decrease in estimated glomerular filtration rate [eGFR] during treatment), within-patient eGFR slope, and side effects were monitored during the 3-year follow-up.

**Results:**

Forty patients participated in the study. OsmU remained below 200 mOsm/kg throughout the study period, and most patients required the minimum tolvaptan dose (mean dose, 64 [±10] mg), with a low discontinuation rate (5%). The mean annual decline in eGFR was -3.05 (±2.41) ml/min per 1.73 m^2^ during tolvaptan treatment, compared to the period preceding treatment, corresponding to a reduction in eGFR decline of more than 50%. Primary renal events occurred in 20% of patients (mean time to onset, 31 months; 95% confidence interval [CI] = 28–34).

**Conclusion:**

Individualized tolvaptan dose adjustment based on OsmU in patients with ADPKD and rapid disease progression provided benefits in terms of reducing eGFR decline, compared with reference studies, and displayed lower dropout rates and fewer side effects. Further studies are required to confirm optimal strategies for the use of OsmU for tolvaptan dose adjustment in patients with ADPKD.

## Graphical abstract

**Figure:**
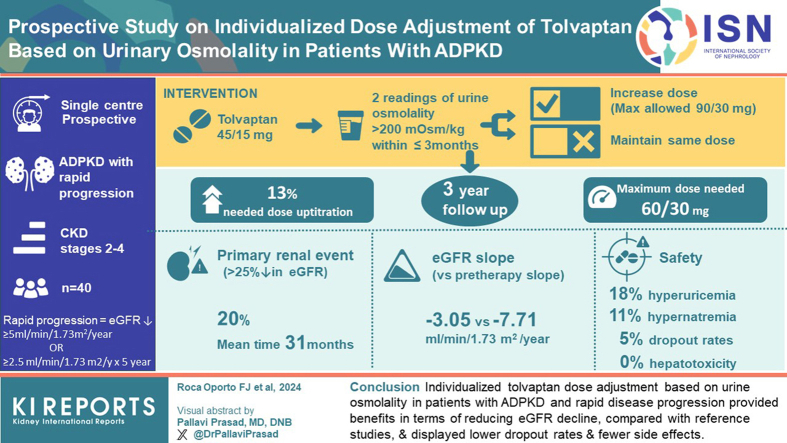

See Commentary on Page 737

ADPKD is the most common hereditary renal disease, with an estimated prevalence of 4 cases per 10,000 people.^1^ This condition is characterized by the development and progressive growth of multiple cysts in the kidneys, leading to the distortion of the renal parenchyma and impaired kidney function.^2^ Arginine vasopressin, the key hormone implicated in cystogenesis, regulates cyclic adenosine monophosphate levels through vasopressin 2 receptors and represents a major therapeutic target for ADPKD.^3^

Tolvaptan, a selective vasopressin antagonist that blocks binding to the vasopressin 2 receptor, is the only available treatment for reducing renal volume and delaying disease progression, as evidenced by the outcomes of the Tolvaptan Efficacy and Safety in Management of Polycystic Kidney Disease and its Outcomes (TEMPO) 3:4^4^ and Replicating Evidence of Preserved Renal Function: An Investigation of Tolvaptan Safety and Efficacy in ADPKD (REPRISE)^5^ studies. Owing to its pharmacokinetic profile,^6^ tolvaptan is administered on a split schedule and is available in 3 doses (45/15 mg, 60/30 mg, and 90/30 mg). The current recommendations suggest a gradual escalation (weekly or monthly) upto the maximum tolerated dose, depending on the tolerance of the patient to aquaretic effects and hepatotoxicity.^7^ Notably, the tolerability of tolvaptan decreases significantly at high doses,^8^ leading to more aquaretic effects such as pollakiuria and thirst. Therefore, the identification of suitable biomarkers to help guide the tolvaptan dose adjustment is required.

OsmU has been studied as a potential biomarker for dose adjustment because OsmU is an indicator of vasopressin 2 receptor blockade. However, the extent of receptor suppression at different doses of tolvaptan remains unresolved.^9^ Therefore, we aimed to explore the possibility of individualized dose adjustment of tolvaptan according to OsmU in patients with ADPKD, with rapid disease progression. We assessed the feasibility of OsmU as a biomarker for individualized dose adjustment of tolvaptan in patients with this condition.

## Methods

### Study Design and Population

This single-center prospective cohort study was conducted at the Hospital Universitario Virgen del Rocío in Seville, Spain; and included patients with ADPKD who met the criteria for rapid disease progression and received tolvaptan treatment between January 1, 2017, and December 31, 2021. Patients aged ≥18 years with ADPKD at stages 2 to 4 of chronic kidney disease (CKD) were included in this study. Rapid disease progression was defined as a decrease in the eGFR ≥5 ml/min per 1.73 m^2^ in the preceding year or ≥2.5 ml/min per 1.73 m^2^ per year during the previous 5 years.

All participants provided written informed consent in accordance with the research protocol, which was approved by the Hospital Research Ethics Committee, and conformed with the principles of the Declaration of Helsinki and the Declaration of Istanbul.

### Drug Exposure

The efficacy of tolvaptan has been defined as the ability to maintain OsmU below 280 to 300 mOsm/kg (hypotonic urine relative to plasma)^10^ measured in the first predose morning urine.^8^ In the TEMPO 3:4 *post hoc* study, the second morning void was used for spot urine osmolarity measurements and did not reveal significant benefits of reducing OsmU below 250 mOsm/kg. However, patients with a higher degree of OsmU suppression from baseline experienced a reduction in clinical events.^11^ In a tolvaptan pharmacokinetics and pharmacodynamics study, a 90/30 mg dose helped maintain the OsmU below 200 mOsm/kg throughout the day, except in the morning OsmU measurement in 15% of patients.^6^ Based on these findings, we established a cut-off point of 200 mOsm/kg for dose adjustment. The efficacy of tolvaptan was evaluated by measuring the slope of eGFR decrease (ml/min per 1.73 m^2^ per year), with individual dose adjustment according to OsmU and the limit set at 200 mOsm/kg. Patients were initially treated with tolvaptan doses of 45/15 mg, with a dose increase if OsmU was >200 mOsm/kg in 2 consecutive determinations within <3 months. Patients were followed-up with for 3 years.

### Data and Sample Collection and Analytical Methods

Blood and urine samples were collected in the morning on an empty stomach before administering the first drug dose. Analyses were performed before starting treatment—every month during the first 18 months of treatment and every 3 months thereafter. Twenty-four-hour OsmU, measured using a freezing point osmometer, was analyzed using a standard laboratory technique. Proteinuria and urinary density were measured using the first morning urine sample. The eGFR was estimated using the Chronic Kidney Disease Epidemiology Collaboration equation based on serum creatinine, 2021 Chronic Kidney Disease Epidemiology Collaboration formulation for eGFR.^12^ The mean annual decrease in eGFR was calculated using the eGFR at 12, 24, and 36 months of treatment with respect to the baseline eGFR. When available, information on renal size was obtained using magnetic resonance imaging or computed tomography, and total kidney volume (TKV) was measured before and after treatment. The TKV was corrected for height (height-adjusted TKV) and age to determine the Mayo class.^13^

### Outcomes

The primary end point was disease progression, defined as a 25% decrease in eGFR during treatment, compared with baseline and within-patient eGFR slope (analyze the values of eGFR across time depending on the dose of tolvaptan). The secondary end point corresponded to the following series of composite events over time: episodes of complicated cysts requiring medical intervention, worsening of albuminuria (with a change of category), the onset of hypertension, or the need for increased treatment for hypertension. Diuretics were permitted in this study.

Drug side effects such as diuresis, glycemia, natremia, uric acid, medication withdrawal, and hepatotoxicity—defined as alanine aminotransferase, aspartate aminotransferase, or γ-glutamyl transferase levels >3 times the normal value, and total bilirubin levels >2 times the normal value—were also recorded. Treatment was discontinued when the patient reached an eGFR of <20 ml/min per 1.73 m^2^.

### Statistical Analyses

Descriptive analyses were performed for all variables collected. The normality of data distribution was assessed using the Kolmogorov–Smirnov test. The changes in eGFR were analyzed using repeated measures analysis of variance, and the assumption of sphericity was evaluated using Mauchly's W statistic, with its statistical significance considered due to the violation of this assumption. Subsequently, Bonferroni *post hoc* tests were performed to determine differences between 2 time points. The effect size was calculated using a partial E-square. Survival free of primary and secondary renal events was analyzed separately using the Kaplan–Meier method. The occurrence of the corresponding renal event was considered the cut-off point for treatment termination, and the time variable was measured in months until the event occurred. Subsequently, primary renal event-free survival was analyzed according to the degree of renal disease and tolvaptan dose using the Kaplan–Meier method, with grouping variables treated as factors. Comparisons between factor levels were performed using the log-rank test (Mantel–Cox). Primary and secondary Cox regression models were used to determine the factors associated with survival-free renal events. In addition, mixed analysis of variance were carried out to analyze the intergroup (tolvaptan dose 45/15 mg vs. tolvaptan dose 60/30 mg) and intr-group (over time) effect of tolvaptan on eGFR. The lower-bound correction was applied given the results of the hypothesis contrasts testing the required assumptions for this analysis. The analyses were performed using IBM SPSS v.29 (IBM, Armonk, NY) statistical packages. R software 3.6.2 (R Foundation for Statistical Computing, Vienna, Austria) was used to prepare figures.

## Results

### Population Characteristics

**Figure 1:**
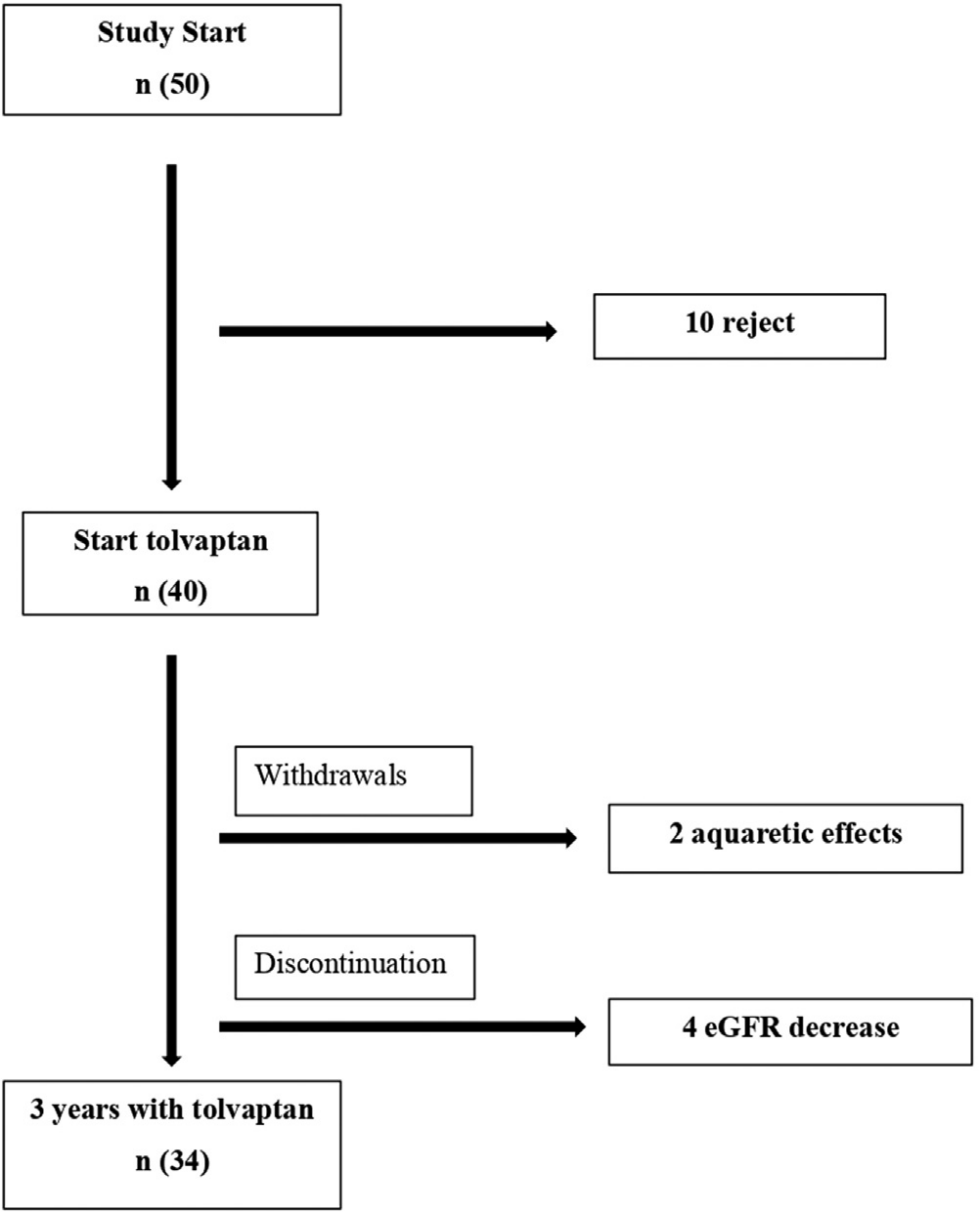
Flow chart of patient enrolment. eGFR, estimated glomerular filtration rate.

In our center, there were only 50 eligible patients that met the study criteria; 40 consented to participate in the study. In Table 1, we present the baseline patient characteristics. Forty-seven percent were men, with a mean age of 45 (±7) years. The baseline eGFR was 51.05 (± 12.52) ml/min per 1.73 m^2^. Most patients had hypertension (82%) and were receiving treatment for that condition. The patients were followed-up with for an average of 34 (±4) months, with 85% (34/40) completing 3 years of tolvaptan treatment. Two patients (5%) dropped out within the first year because of aquaretic effects, and tolvaptan was discontinued in 4 patients (10%) who had been on treatment for over 2 years, due to eGFR falling below 20 ml/min per 1.73 m^2^ (Figure 1).

**Table 1:** Baseline demographic and clinical characteristics of adolescents undergoing treatment with tolvaptan at the start of the study

Demographic and clinical characteristics at baseline	Values
Age (yr), mean±SD	45 ± 7
Sex (male), *n* (%)	19 (47)
Weight (kg), mean ± SD	77 ± 17
Height (cm), mean ± SD	172 ± 9
Body mass index (kg/m^2^), mean ± SD	26 ± 4.5
Estimated GFR, ml/min per 1.73 m^2^, mean ± SD	51.05 ± 12.52
Chronic kidney disease stage, *n* (%)	
1	0 (0)
2	9 (22)
3	31 (78)
3a	18 (58)
3b	13 (42)
4	0 (0)
Mayo Clinical Classification (36)	
1B	5 (14)
1C	19 (53)
1D	8 (22)
1E	4 (11)
TKV (36) (ml), mean±SD	1785 ± 834
HtTKV (36) (ml/m), mean±SD	1074 ± 554
Urinary albumin-to-creatinine ratio, mg/g, median (Q1–Q3)	22 (67–10)
Hypertension, *n* (%)	33 (82)
Systolic blood pressure, mmHg, mean±SD	138 ± 15
Diastolic blood pressure, mmHg, mean±SD	84 ± 11
RAAS inhibitor treatment, *n* (%)	29 (72)

GFR, glomerular filtration rate; HtTKV, height-adjusted total kidney volume; Q, quartile; RAAS, renin-angiotensin-aldosterone system; TKV, total kidney volume.

### Tolvaptan Exposure

All participants were administered tolvaptan at an initial dose of 45/15 mg. During the follow-up period, the dose was increased based on OsmU in 5 patients (13%), with tolvaptan titration reaching 60/30 mg (Table 2). None of the patients received a dose of 90/30 mg. The mean tolvaptan dose administered was 64 (±10) mg.

**Table 2:** Annual doses of tolvaptan and eGFR

Variables	Treatment period				
	Baseline *N* = 40	Month 1 *N* =39	Year 1 *N* = 38	Year 2 *N* = 38	Year 3 *N* = 34
eGFR, ml/min per 1.73 m^2^, mean±SD	51.03 ± 12.53	49.85 ± 12.56	49.15 ± 13.69	45.18 ± 14.70	43.03 ± 13.40
Tolvaptan dose (mg), mean (SD)	60 (40)	60 (40)	60 (35)	60 (33)	60 (30)
	90 (0)	90 (0)	90 (3)	90 (5)	90 (4)
Urine osmolality (mOsm/Kg), mean±SD	391 ± 87	171 ± 53	173 ± 45	163 ± 33	162 ± 39
Urinary albumin-to-creatinine (mg/g), median (Q1–Q3)	22 (67–10)	10 (8–42)	10 (5–61)	16 (5–50)	20 (8–41)

	Nontreatment period				
		3 years *N* = 40	2 years *N* = 40	1 year *N* = 40	
eGFR, ml/min per 1.73 m^2^, mean±SD		73.97 ± 17.57	67.57 ± 15.08	59.63 ± 16.44	

	Slope of the annual decrease in eGFR				
		No tolvaptan		Tolvaptan	
eGFR, ml/min per 1.73 m^2^, mean±SD		−7.71 ± 4.15		−3.05 ± 2.41	

	Kidney volume, *n* = 12				
		Baseline		Tolvaptan treated	
TKV (ml), mean ± SD		1591 ± 860		1787 ± 884	
HtTKV (ml/m), mean ± SD		914 ± 486		1033 ± 505	

eGFR, estimated glomerular filtration rate; HtTKV, height-adjusted total kidney volume; Q, quartile; TKV, total kidney volume.

### Urine Osmolality

**Figure 2:**
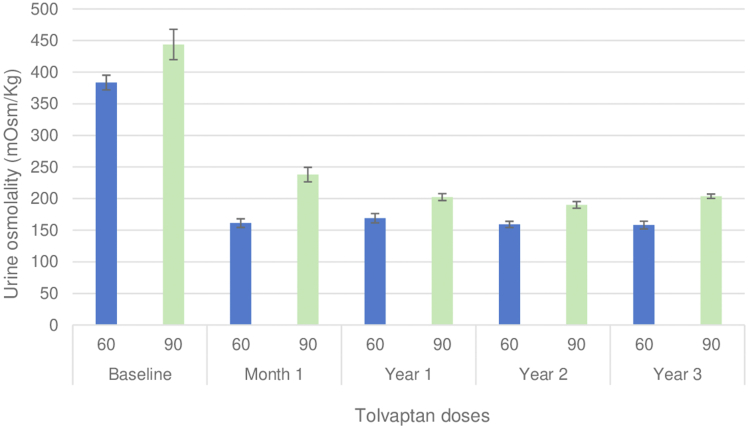
Tolvaptan doses and urine osmolality during follow-up. Patients were administered tolvaptan with adjusted doses based on urinary osmolality during the 36-month period. Urine osmolality: 24-hour measurement.

Over 3 years, the mean 24-hour OsmU was 166 (±30) mOsm/kg. The most significant change occurred within the first month, with a difference of -220 mOsm/kg (95% CI = −188 to −255) compared with that at baseline (Table 2). Baseline OsmU demonstrated a positive correlation with baseline eGFR (r = 0.30; *P* = 0.014) (Supplementary Table S1). There were no significant differences in the patients receiving a 60/30 mg tolvaptan baseline OsmU in the first month and throughout the 3-year treatment compared with those receiving a 45/15 mg dose (*P* > 0.05) (Figure 2; Supplementary Table S2).

### Renal Events

**Figure 3:**
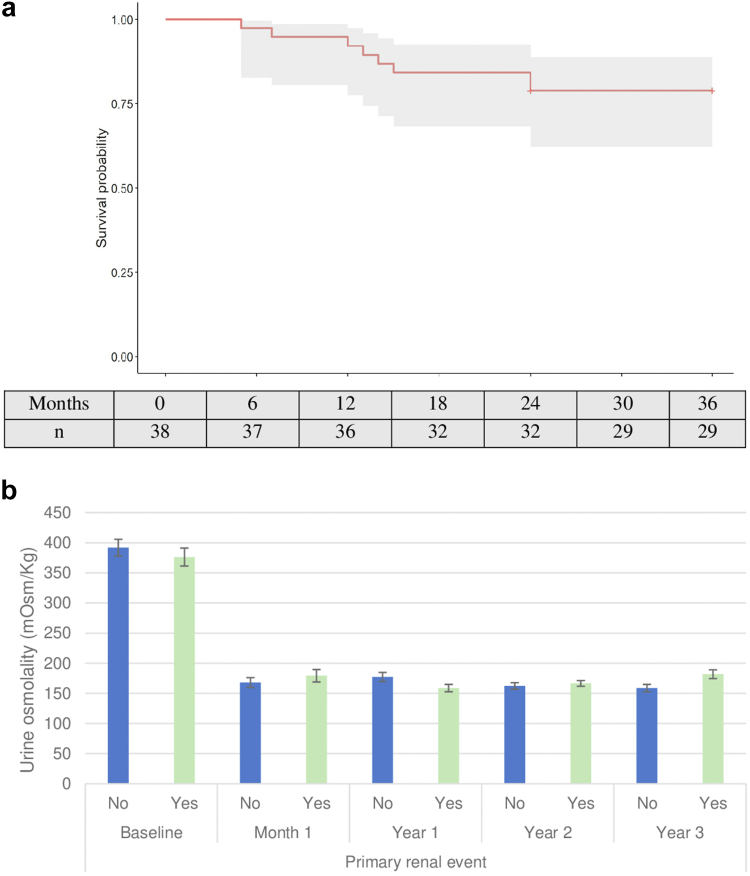
(a) Survival curve for the occurrence of the primary renal event. Kaplan–Meier estimate of primary renal events, a 25% decrease in eGFR from baseline, in the study cohort. (b) Urine osmolality and primary renal events during the 36-month period. Urine osmolality: 24-hour measurement.

During the 3-year period, the mean annual decline in eGFR was −3.05 (±2.41) ml/min per 1.73 m^2^ with treatment, whereas the observed decline before treatment was −7.71 (±4.15) ml/min per 1.73 m^2^ (*P* < 0.001) (Table 2). A primary renal event, corresponding to a 25% decrease in eGFR from baseline, occurred in 8 patients (20%), with a mean time to onset of 31 months (95% CI = 28; 34) (Figure 3a). The OsmU did not significantly differ between patients without primary renal events and those with primary renal events during the 3 years (171 ± 9 vs. 166 ± 7 mOsm/kg) (*P* > 0.05) (Figure 3b; Supplementary Table S3). According to the CKD stage, 9 patients were in stage 2, none of whom presented with a primary renal event; 18 patients were in stage 3a and 3 of them (17%) presented with an event; 13 patients were in stage 3b, of whom 5 (38%) presented with an event. Regarding the tolvaptan dose, 35 patients had a final dose of 45/60 mg, among whom 6 (17%) presented with a primary renal event; 5 patients had a final dose of 60/30 mg, among whom 2 (40%) presented with an event (*P* > 0.05). In survival analysis according to CKD stage (*P* = 0.081) and tolvaptan dose (*P* = 0.20), no significant differences were found in primary renal events (Supplementary Tables S4 and S5).

In the analysis within-patient eGFR, there were not differences in the values of eGFR across time depending on the dose of tolvaptan (*P* = 0.10) (Supplementary Table S6)

**Figure 4:**
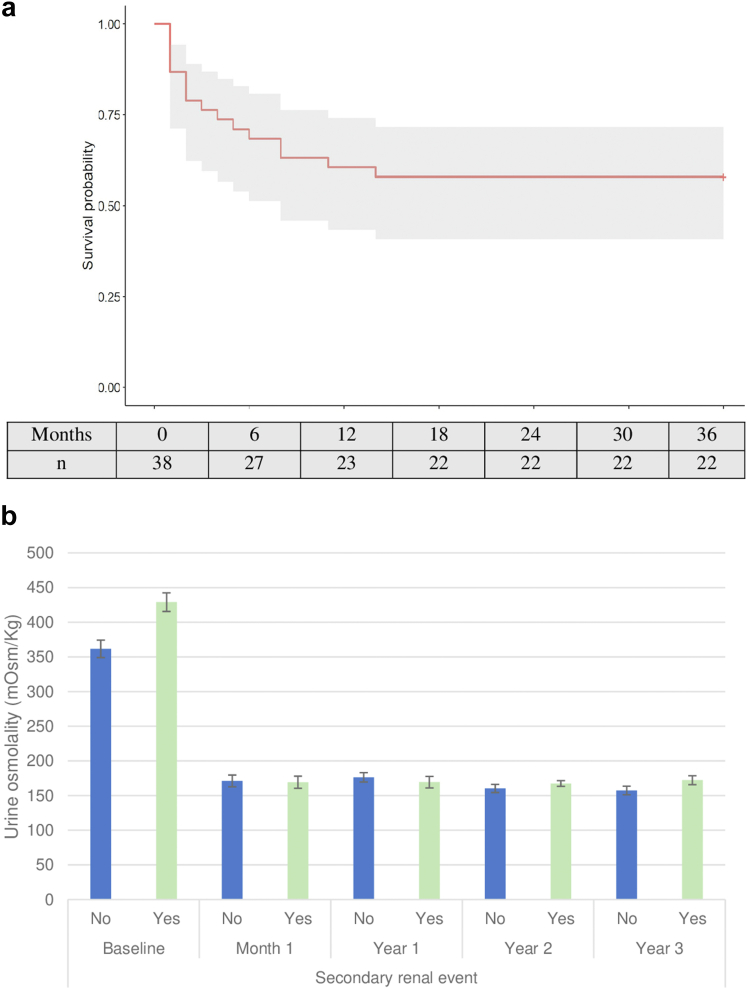
(a) Survival curve of secondary renal event occurrence. Kaplan–Meier estimate of secondary renal events in the study cohort. (b) Urine osmolality and secondary renal events during the 36-month period. Urine osmolality: 24-hour measurement.

A total of 16 patients (40%) presented with secondary renal events, with a mean time to onset of 21 months (95% CI = 16; 27) (Figure 4a). The most frequent complication was poor control of hypertension (22%), followed by episodes of complicated cysts (e.g., ruptured and infected) (Supplementary Table S7). Patients with secondary renal events had a higher baseline OsmU than those without (429 ± 85 vs. 361 ± 80 mOsm/kg) (*P* = 0.019). During the 3-year treatment period, there was no significant difference in OsmU between the 2 groups (166 ± 8 vs. 169 ± 6 mOsm/kg) (*P* > 0.05) (Figure 4b; Supplementary Table S8). In the multivariate analysis of factors associated with renal event-free survival, baseline eGFR was observed to be a protective factor for both primary (hazard ratio: 0.88; 95% CI = 0.807–0.971) and secondary (hazard ratio 0.92; 95% CI = 0.871–0.969) renal events (Supplementary Tables S9 and S10).

### TKV

Of the 40 patients receiving treatment, only 12 had TKV data both at the baseline and at the end of the treatment. During the 3-year treatment period, renal volume increased by 3.5% annually.

### Side Effects

The most frequent side effects observed were hyperuricemia (18%), requiring treatment in 10% of patients; and hypernatremia (11%), with a maximum value of 148 mEq/l (Table 3). No cases of hepatotoxicity were reported. Polyuria was recorded in all patients, with a median diuresis of 6000 ml/day (5500–7000); 2 patients dropped out due to aquaretic effects (5%).

**Table 3:** Summary of adverse events during tolvaptan treatment

Adverse Events during the Study Period	Values
Adverse events	
Diuresis (ml/d), median (Q1–Q3)	6000 (7000–5500)
Serum uric acid (mg/dl), median (Q1–Q3)	6.3 (5.4–7.2)
Serum uric acid > 7.5 mg/dl, *n* (%)	7 (18)
Treatment hyperuricemia, *n* (%)	4 (10)
Serum glucose (mg/dl), mean±SD	85 ± 9,6
Serum sodium (mEq/l), median (Q1–Q3)	142 (141–144)
Hypernatremia > 145 mEq/l, *n* (%)	4 (11)
Hypernatremia > 150 mEq/l, *n* (%)	0 (0)
Liver enzymes alanine aminotransferase (U/l), median (Q1–Q3)	19 (22–16)
aspartate aminotransferase (U/l), median (Q1–Q3)	17 (22–13)
g-glutamyltransferase (UI/l), median (Q1–Q3)	21 (34–14)
total bilirubin (mg/dl), median (Q1–Q3)	0.3 (0.5–0.3)
Liver injury, *n* (%)	0 (0)
Serum alanine aminotransferase >3 times upper limit of normal	0 (0)
Serum aspartate aminotransferase >3 times upper limit of normal	0 (0)
Serum total bilirubin >2 times upper limit of normal	0 (0)
Discontinuation related to adverse events, *n* (%)	2 (5)
Treatment discontinuation eGFR <20 ml/min per 1.73 m^2^, *n* (%)	4 (10)

eGFR, estimated glomerular filtration rate; Q, quartile.

## Discussion

We explored the feasibility of OsmU as a biomarker for individualized tolvaptan dose adjustment in patients with ADPKD. Our findings reveal that patients undergoing tolvaptan treatment presented OsmU below 200 mOsm/kg during the 3-year follow-up period, with most patients being administered the minimum dose of tolvaptan. This was a fundamental difference compared with large pivotal studies, such as TEMPO 3:4^4^ and REPRISE,^5^ in which the maximum dose of the drug was predominantly used. A systematic review and meta-analysis of 8 studies revealed a lack of evidence regarding the efficacy of treatment with different doses of tolvaptan.^14^ In ADPKD, even in the early stages of the disease, there is a defect in the ability to concentrate urine,^15^ due to cystic damage to the medullary gradient. In our study, the baseline OsmU (391 ± 87 mOsm/kg) was lower than that reported in large trials like TEMPO 3:4 (504 ± 177 mOsm/kg).^11^ This lower baseline OsmU may explain the need for lower tolvaptan doses in our study, particularly considering the initial response to the drug. We observed that patients who required dose increases during follow-up had higher baseline OsmU.

We evaluated the activity of tolvaptan based on the slope of the decrease in eGFR. Despite using lower tolvaptan doses (45/15 mg), the annual decrease in eGFR was reduced by more than 50%. A meta-analysis^16^ of 13 randomized studies of tolvaptan showed a mean difference in eGFR of 1.27 ml/min per 1.73 m^2^ per year between treatment and placebo groups (95% CI: 1.24–1.29). In our study, the renal volume growth with tolvaptan was less than 5% per year, which is below the limit set by international guidelines and recommendations for classification as rapid disease progression.^17^^,^^18^ We could not confirm whether enhanced suppression of OsmU correlated with a better prognosis of renal events. Furthermore, the analysis of different tolvaptan doses (45/15 and 60/30 mg) and CKD stages (2 and 3) did not reveal significant differences between the groups. Previous studies have described a milder decline in eGFR among patients with preserved renal function.^19^ In this study, the only protective factor identified was baseline eGFR, consistent with previous research,^18^ indicating that patients with preserved renal function and rapid disease progression benefit more from treatment with tolvaptan in the early stages of CKD (stage 1–3).^10^

The main side effects of tolvaptan are thirst, polyuria, hyperuricemia, and hepatic alterations with elevated transaminases.^20^ In this study, all patients experienced polyuria, despite being administered a minimal tolvaptan dose, and 2 patients (5%) discontinued treatment because of aquaretic effects in the first year. High doses of tolvaptan are associated with increased side effects, leading to a higher dropout rate,^8^ especially in patients with early-stage CKD.^21^ In previous studies, the observed dropout rates varied as follows: 23% in TEMPO 3:4,^20^ 22% in a 1-year retrospective cohort from Andalusia,^22^ and 56% in a recent 3-year retrospective cohort study from the United Kingdom.^23^ The use of the maximum tolvaptan dose could contribute to these undesirable effects. Notably, we did not observe any cases of hepatotoxicity in this study.

This study had several limitations. First, it was not randomized and lacked a control group, limiting the ability to establish direct comparisons. Second, tolvaptan dosage was increased in only 5 patients, leaving no option to reliably perform between-patients comparison. Third, we could not assess TKV during renal events. Although TKV^24^ has been identified as the main marker of ADPKD progression in large clinical trials,^25^ routine serial use of computed tomography or magnetic resonance imaging was not part of the usual clinical management of these patients. Fourth, the eGFR slope of −7.71 ml/min per 1.73 m^2^ per year before treatment is very steep compared to other nontreated patients in the TEMPO and REPRISE studies; thus, we cannot exclude the possibility that the rapid eGFR decline in this group is caused by regression to the mean and not by tolvaptan only. Fifth, did not measure plasma copeptin levels, a marker of plasma vasopressin. It has been shown that, it can predict disease progression and tolvaptan efficacy in ADPKD.^26^ Lastly, the number of patients from a single center in our study was smaller than that in larger studies. Nonetheless, to the best of our knowledge, this is the first report of OsmU used as a biomarker for adjusting the tolvaptan dose in patients with ADPKD. The major strengths were the duration of our study, which included a 3-year prospective follow-up, and the documented history of eGFR before tolvaptan treatment. The homogeneous criteria for indication and management, stemming from the single-center design, also contribute to the reliability of the study.

In conclusion, in this prospective study on adult patients with ADPKD and rapid disease progression, we found that OsmU suppression below 200 mOsm/kg, was associated with a decrease in renal disease progression. This resulted in the use of lower tolvaptan doses, a lower dropout rate, and fewer side effects. Larger prospective randomized controlled studies are necessary to determine the potential role of OsmU-based dose titration of tolvaptan as an alternative in clinical practice for patients with ADPKD and rapid disease progression.

## Disclosure

All the authors declared no competing interests.

## Supplementary Material

Supplementary File (PDF)

**Table S1**. Correlation between urine osmolality and estimated glomerular filtration rate.

**Table S2**. Urine osmolality and tolvaptan doses.

**Table S3**. Primary renal event and urine osmolality.

**Table S4**. Primary renal event-free survival by degree of kidney disease.

**Table S5**. Primary renal event-free survival according to tolvaptan dose.

**Table S6.** Mixed analysis of variance: estimated glomerular filtration rate across time depending on the dose of tolvaptan.

**Table S7.** Primary and secondary renal events.

**Table S8**. Secondary renal event and urine osmolality.

**Table S9.** Factors associated with primary renal event-free survival.

**Table S10.** Factors associated with secondary renal event-free survival.

STROBE statement
