# Supplementary material for: Prospective Study on Individualized Dose Adjustment of Tolvaptan Based on Urinary Osmolality in Patients With ADPKD
Source: Kidney Int Rep. 2024 Jan 12;9(4):1031–9. doi: 10.1016/j.ekir.2024.01.020 (PMC11101827; doi:10.1016/j.ekir.2024.01.020)
Supplement: Supplementary File (PDF) [file mmc1.pdf]

Supplementary Material

Supplemental Table S1. Correlation between urine osmolality and eGFR.

Supplemental Table S2. Urine osmolality and tolvaptan doses.

Supplemental Table S3. Primary renal event and urine osmolality.

Supplemental Table S4. Primary renal event-free survival by degree of kidney disease.

Supplemental Table S5. Primary renal event-free survival according to tolvaptan dose.

Supplemental Table S6. Mixed ANOVAS: eGFR across time depending on the dose of tolvaptan.

Supplemental Table S7. Primary and secondary renal events.

Supplemental Table S8. Secondary renal event and urine osmolality.

Supplemental Table S9. Factors associated with primary renal event-free survival.

Supplemental Table S10. Factors associated with secondary renal event-free survival.

STROBE statement (PDF)

Supplemental Table S1. Correlations between urine osmolality throughout follow-up and eGFR.

eGFR	Urine osmolality									
	Baseline		1 month treatment		1 year treatment		2 years treatment		3 years treatment	
	r	p-value	r	p-value	r	p-value	r	p-value	r	p-value
3 previous years	**0.412**	**0.015**	-0.234	0.175	-0.152	0.392	0.069	0.697	-0.017	0.930
2 previous years	**0.489**	**0.002**	-0.047	0.779	0.089	0.602	0.274	0.101	0.110	0.556
1 previous year	**0.515**	**0.001**	-0.087	0.598	0.081	0.630	0.186	0.264	-0.005	0.979
Baseline	**0.389**	**0.014**	-0.148	0.368	0.096	0.566	0.059	0.726	-0.115	0.530
1 month treatment	0.211	0.203	-0.218	0.182	0.013	0.940	0.060	0.720	-0.136	0.457
1 year treatment	**0.361**	**0.028**	-0.130	0.435	0.229	0.166	0.190	0.252	0.012	0.946
2 years treatment	**0.389**	**0.017**	-0.092	0.582	0.253	0.125	0.186	0.265	-0.059	0.747
3 years treatment	**0.447**	**0.010**	-0.070	0.700	0.129	0.474	0.073	0.687	-0.026	0.886

r: Pearson's linear correlation coefficient. estimated glomerular filtration rate (eGFR).

Supplemental Table S2. Urine osmolality and tolvaptan doses.

Urine Osmolality (mOsm/Kg)	Tolvaptan doses	Mean	SD	p-value
Basal	60	383.56	73.77	0.150[1]
	90	443.8	151.52	
1 month treatment	60	161.21	43	0.076[2]
	90	238.00	72.69	
1 year treatment	60	168.97	46.76	0.135[1]
	90	202.40	34.66	
2 years treatment	60	159.18	31.38	**0.050[1]**
	90	190.00	33.59	
3 years treatment	60	158.17	38.74	0.057[1]
	90	203.67	22.37	

SD, standard deviation.

1t-Student for independent samples assuming equality of variances by the Levene test.

2t-Student for independent samples without assuming equality of variances by the Levene test.

Supplemental Table S3. Primary renal event and urine osmolality.

Urine Osmolality (mOsm/Kg)	Primary renal event	Mean	SD	p-valor[1]
Baseline	No	391.87	87.05	0.677
	Yes	376.29	94.53	
1 month treatment	No	167.93	51.33	0.604
	Yes	179.25	65.28	
1 year treatment	No	177.27	48.05	0.322
	Yes	158.75	38.71	
2 years treatment	No	162.37	34.09	0.757
	Yes	166.5	30.06	
3 years treatment	No	158.85	38.24	0.240
	Yes	181.8	45.86	

SD: Standard deviation.
1t-Student for independent samples assuming equality of variances by the Levene test.

Supplemental Table S4. Primary renal event-free survival by degree of kidney disease.

Chronic kidney disease stage start		Month	Event	Cumulative proportion that survives over time		Number of cumulative events	Number of remaining cases
				Estimate	Standard error		
2	1	36	0	.	.	0	7
	2	36	0	.	.	0	6
	3	36	0	.	.	0	5
	4	36	0	.	.	0	4
	5	36	0	.	.	0	3
	6	36	0	.	.	0	2
	7	36	0	.	.	0	1
3a	1	14	1	0.941	0.057	1	16
	2	15	1	0.882	0.078	2	15
	3	24	1	0.824	0.092	3	14
	4	36	0	.	.	3	13
	5	36	0	.	.	3	12
	6	36	0	.	.	3	11
	7	36	0	.	.	3	10
	8	36	0	.	.	3	9
	9	36	0	.	.	3	8
	10	36	0	.	.	3	7
	11	36	0	.	.	3	6
	12	36	0	.	.	3	5
	13	36	0	.	.	3	4
	14	36	0	.	.	3	3
	15	36	0	.	.	3	2
	16	36	0	.	.	3	1
	17	36	0	.	.	3	0
3b	1	5	1	0.923	0.074	1	12
	2	7	1	0.846	0.100	2	11
	3	12	1	0.769	0.117	3	10
	4	13	1	0.692	0.128	4	9
	5	24	1	0.615	0.135	5	8
	6	24	0	.	.	5	7
	7	36	0	.	.	5	6
	8	36	0	.	.	5	5
	9	36	0	.	.	5	4
	10	36	0	.	.	5	3
	11	36	0	.	.	5	2
	12	36	0	.	.	5	1
	13	36	0	.	.	5	0

Supplemental Table S5. Primary renal event-free survival according to tolvaptan dose.

Final Tolvaptan Doses		Month	State	Cumulative proportion that survives over time		Number of cumulative events	Number of remaining cases
				Estimate	Standard error		
60	1	5	1	0.970	0.030	1	32
	2	7	1	0.939	0.042	2	31
	3	14	1	0.909	0.050	3	30
	4	15	1	0.879	0.057	4	29
	5	24	1	.	.	5	28
	6	24	1	0.818	0.067	6	27
	7	24	0	.	.	6	26
	8	36	0	.	.	6	25
	9	36	0	.	.	6	24
	10	36	0	.	.	6	23
	11	36	0	.	.	6	22
	12	36	0	.	.	6	21
	13	36	0	.	.	6	20
	14	36	0	.	.	6	19
	15	36	0	.	.	6	18
	16	36	0	.	.	6	17
	17	36	0	.	.	6	16
	18	36	0	.	.	6	15
	19	36	0	.	.	6	14
	20	36	0	.	.	6	13
	21	36	0	.	.	6	12
	22	36	0	.	.	6	11
	23	36	0	.	.	6	10
	24	36	0	.	.	6	9
	25	36	0	.	.	6	8
	26	36	0	.	.	6	7
	27	36	0	.	.	6	6
	28	36	0	.	.	6	5
	29	36	0	.	.	6	4
	30	36	0	.	.	6	3
	31	36	0	.	.	6	2
	32	36	0	.	.	6	1
	33	36	0	.	.	6	0
90	1	12	1	0.800	0.179	1	4
	2	13	1	0.600	0.219	2	3
	3	36	0	.	.	2	2
	4	36	0	.	.	2	1
	5	36	0	.	.	2	0

Supplemental Table S6. Mixed ANOVAS: eGFR across time depending on the dose of tolvaptan.

Effect	p-value
Intra-group	<0.001
Inter-group	0.103
Interaction	0.209

Supplemental Table S7. Primary and secondary renal events.

Variable	Category	n	%
Primary renal event (n 38)	No	30	78.9
	Yes	8	21.1
Primary renal event months (n 38)	Mean (DE)	14.25 (6.92)	
Secondary renal events (n 38)	No	22	57.9
	Yes	16	42.1
Secondary renal events months (n 16)	Mean (DE)	22 (4.02)	
Worsening albuminuria (n 38)	No	37	97.4
	Yes	1	2.6
Hypertension (onset or treatment) (n 38)	No	30	78.9
	Yes	8	21.1
Complicated cyst (n 38)	No	31	81.6
	Yes	7	18.4
SD: Standard deviation.			

Supplemental Table S8. Secondary renal events and urine osmolality.

Urine osmolality (mOsm/Kg)	Secondary renal event	Mean	SD	p-valor[1]
Baseline	No	361.59	79.63	**0.019**
	Yes	429	84.96	
1 month treatment	No	171.09	53.87	0.919
	Yes	169.25	55.42	
1 year treatment	No	176.27	42.35	0.657
	Yes	169.38	52.61	
2 years treatment	No	160.23	37.52	0.516
	Yes	167.38	25.96	
3 years treatment	No	157.33	39.1	0.322
	Yes	172.18	40.61	

SD: Standard deviation.
1t-Student for independent samples assuming equality of variances by the Levene test.

Supplemental Table S9. Factors associated with primary renal event-free survival.

Variable	HR	IC95%(HR)	p-value
Age	0.864	(0.742;1.005)	0.059
eGFR baseline	0.885	(0.807;0.971)	0.010

*Reference category.

HR: Hazard Ratio; CI: Confidence interval; eGFR: estimated glomerular filtration rate.

-2*Logarithm of likelihood = 39.581; Chi-square=8.016(2gl), p=0.018

Supplemental Table S10. Factors associated with secondary renal event-free survival.

Variable	HR	IC95%(HR)	p-value
eGFR baseline	0.919	(0.871;0.969)	0.002
OsmU baseline	1.010	(1.003;1.016)	0.003

*Reference category.
HR: Hazard Ratio; CI: Confidence interval; eGFR: estimated glomerular filtration rate.
-2*Logarithm of likelihood=84.967; Chi-square=17.351(2gl), p<0.001

STROBE Statement—Checklist of items that should be included in reports of ***cohort studies***

	Item No	Recommendation	Page No
**Title and abstract**	1	(*a*) Indicate the study's design with a commonly used term in the title or the abstract	1-2
		(*b*) Provide in the abstract an informative and balanced summary of what was done and what was found	2
**Introduction**			
Background/rationale	2	Explain the scientific background and rationale for the investigation being reported	4
Objectives	3	State specific objectives, including any prespecified hypotheses	4-5
**Methods**			
Study design	4	Present key elements of study design early in the paper	6-9
Setting	5	Describe the setting, locations, and relevant dates, including periods of recruitment, exposure, follow-up, and data collection	4-7
Participants	6	(*a*) Give the eligibility criteria, and the sources and methods of selection of participants. Describe methods of follow-up	4
		(*b*) For matched studies, give matching criteria and number of exposed and unexposed	n/a
Variables	7	Clearly define all outcomes, exposures, predictors, potential confounders, and effect modifiers. Give diagnostic criteria, if applicable	5-7
Data sources/ measurement	8*	For each variable of interest, give sources of data and details of methods of assessment (measurement). Describe comparability of assessment methods if there is more than one group	4-7
Bias	9	Describe any efforts to address potential sources of bias	9
Study size	10	Explain how the study size was arrived at	6
Quantitative variables	11	Explain how quantitative variables were handled in the analyses. If applicable, describe which groupings were chosen and why	5
Statistical methods	12	(*a*) Describe all statistical methods, including those used to control for confounding	5
		(*b*) Describe any methods used to examine subgroups and interactions	5
		(*c*) Explain how missing data were addressed	Figure 1
		(*d*) If applicable, explain how loss to follow-up was addressed	Figure 1

		(*e*) Describe any sensitivity analyses	n/a
**Results**			
Participants	13*	(a) Report numbers of individuals at each stage of study—eg numbers potentially eligible, examined for eligibility, confirmed eligible, included in the study, completing follow-up, and analysed	6, Figure1
		(b) Give reasons for non-participation at each stage	6
		(c) Consider use of a flow diagram	Figure 1
Descriptive data	14*	(a) Give characteristics of study participants (eg demographic, clinical, social) and information on exposures and potential confounders	Table 1-2
		(b) Indicate number of participants with missing data for each variable of interest	Figure 1
		(c) Summarise follow-up time (eg, average and total amount)	6-7, Table2
Outcome data	15*	Report numbers of outcome events or summary measures over time	6-7
